# An exploration of nursing students' lived experience of clinical placement in adult male prisons: A phenomenological study

**DOI:** 10.1111/jan.16355

**Published:** 2024-07-29

**Authors:** Joanne Brooke, Monika Rybacka, Shubhangi Sarwan, Omorogieva Ojo

**Affiliations:** ^1^ Centre of Social Care, Health, and Related Research Birmingham City University Birmingham UK; ^2^ School of Nursing, Midwifery and Health Coventry University Coventry UK; ^3^ School of Health Sciences University of Greenwich London UK

**Keywords:** education, nursing, prisons, research, students, nursing

## Abstract

**Aim:**

To explore nursing students' lived experience of a clinical placement within healthcare in a prison, to gain an insight into the support provided prior to and during this unique clinical placement.

**Design:**

An inductive phenomenological study.

**Methods:**

Participants included 14 nursing students from three undergraduate nursing programmes in England, Adult (*n* = 4), Learning Disability (*n* = 3) and Mental Health (*n* = 7). Following a clinical placement in a prison, each participant completed a semistructured audio‐recorded interview on MS Teams between October and December 2021. Audio recordings were transcribed verbatim and thematic analysis was completed.

**Results:**

Two overarching themes were identified, ‘shock’ due to the (a) reality of prison; (b) overwhelming emotional impact and (c) frequency and severity of self‐harm and ‘surprise’ due to (a) the need to work with prison officers; (b) recognizing and addressing preconceptions of people in prison and (c) the development of clinical knowledge, skills and becoming a nurse.

**Conclusions:**

The need remains for a comprehensive strategy of preparation and orientation for nursing students before commencing a clinical placement in prison, which includes the development of knowledge and clinical skills to support the complex health and social care needs of people in prison.

**Impact:**

Our research identified the support provided to nursing students prior to clinical placement in prison varies considerably. The development of a preparation and orientation programme has the potential to reduce pre‐placement anxiety, emotional burden and support nursing students in addressing their preconceptions of people in prison. This approach is essential to support future nursing students to embrace the unique opportunity of a clinical placement within a prison, enhance their clinical knowledge and skills, and develop as a nurse.

**Reporting Method:**

Our paper adheres to the consolidated criteria for reporting qualitative research (COREQ).

**Patient or Public Contribution to the Development of this Study:**

None.

## 1 INTRODUCTION

Globally, the social, economic and environmental disadvantages experienced by people in prison and their lack of engagement in healthcare services increase their risk of illness and the development of complex physical and mental health conditions (Stürup‐Toft et al., 2018). The health of people in prison is significantly impacted by the prison environment, this has been defined as accelerated ageing and acknowledged as a phenomenon for over two decades (Grant, 1999). Accelerated ageing occurs due to the advancement of the physical health of a person in prison by 10 years beyond their chronological age (Grant, 1999). People in prison are significantly more likely to be diagnosed with a blood‐borne virus, substance misuse and/or a mental health disorder than the general population (Baranyi et al., 2022; Singh et al., 2022) and have a higher risk of developing and experiencing an exacerbation of a long‐term condition (LTC) (Massoglia, 2015). People in prison have a higher prevalence of LTCs than those living in the community including diabetes, liver disease, cardiovascular diseases, respiratory and mental health (Favril et al., 2024; Skarupski et al., 2018).

Due to the complex physical and mental health conditions of people in prison, the provision of and access to primary care by people in prison is ‘of utmost importance’ (Groenewegen et al., 2022). The provision of primary care for those in prison is guided by international human rights, such as the United Nations Standard Minimum Rules for the Treatment of Prisoners, referred to as the Nelson Mandela Rules, which identifies people in prison should have access to the same standards of health care that is available in the community, without either discrimination or a monetary charge (UN Office on Drugs and Crime, 2015). However, the governing of primary care within prisons varies considerably, in Australia both state and territory governments are responsible (Australian Institute of Health and Welfare, 2023). Whereas, in Europe, the Ministry of Justice is responsible for prison health in most countries, which is integrated with national care systems (World Health Organization, 2019).

## 2 BACKGROUND

The provision of primary care services within prisons is essential to meet the complex health needs of people in prison and address the health inequalities of this population. In England, the delivery of primary care services within prisons has been supported by local primary care networks (PCNs) (National Health Service [NHS], 2020). The aim of PCNs is the development of ‘proactive, personalised, coordinated, and more integrated health and social care for people close to home’ (NHS England, 2022). The role of the primary care nurse has been defined by the Advanced Clinical Practice (Primary Care Nurses) Framework in 2020 (Royal College of General Practitioners, 2020). The framework supports the development of nurses' skills and expertise, and their recognition within primary care teams. In the prison setting primary care nurses are optimally placed to support the continuity of care of people during their sentence and on release (NHS England & HM Government, 2018).

The role of the primary care nurse commences in the reception of the prison when a person first arrives, and the completion of a holistic assessment, including the person's medical history and social care needs (NHS, 2020). The primary care nurse synthesizes this information to support the referral, treatment, care and support of a person in prison through the development of a personalized care plan (NHS, 2020). Furthermore, primary care nurses develop and implement nurse‐led interventions, such as clinics to support and address sexual health of male adults in prison (Kelly et al., 2020), cardiovascular disease (Bennett, 2014), and hepatitis C virus (Baines, 2022). The advanced skills of primary care nurses and the complex health issues of people in prison identify primary care services in a prison setting as an optimal clinical placement for undergraduate nursing students.

A contemporary review of the literature explored clinical placements and visits to healthcare within a prison, with a focus on how or if nursing students' needs were met to support an optimal learning environment (Brooke et al., 2022). Nursing students identified pre‐placement anxieties due to their own negative attitudes towards people in prison, which influenced their beliefs that this population would be intimidating, abusive and violent (Bouchaud et al., 2017; Hunt et al., 2020). Nursing students' anxieties were exacerbated by their fear of the unknown, which increased when entering prison for the first time (Hunt et al., 2020). During their clinical placements, nursing students identified a sense of safety, an impact on their negative stereotypes and an opportunity for learning (Sutherland et al., 2021; van de Mortel et al., 2017). Further understanding of the anxiety and fear nursing students experienced on commencement of a clinical placement in prison is required to support and prepare future students for this unique learning opportunity. Due to a lack of contemporary research across prisons with different levels of security and prison populations, such as those on remand or serving a prison sentence, there remains a need for a robust study exploring nursing students' experience from different Higher Education Institutes (HEIs), who attend clinical placements in prisons with different levels of security and prison populations.

The aim of this study was to explore nursing students lived experience of a clinical placement within healthcare services in a prison.

## 3 METHODS

### 3.1 Study design

The study design was influenced by inductive phenomenology and the work of Heidegger (1962). Heideggerian philosophy is a theoretical approach and a method that guides the exploration to understand the essence and meaning of being. Heidegger discussed an ontological approach to discover the meaning of being, which is achieved through a process of interpretation of an individual's understanding of their world of objects and relations through language (Heidegger, 1962). Nursing research has applied Heideggerian philosophy, to understand the essence and meaning of particular phenomena, such as receiving or providing healthcare, through exploration of the lived experiences of individuals (Guerrero‐Castañeda et al., 2019). A Heideggerian approach supported the development of this study and the aim to understand nursing students' experience of a clinical placement in prison, and how the students interpreted and made sense of their feelings, provision of care and learning within this restricted environment.

### 3.2 Study setting and recruitment

The setting for our study was primary care health services within the HM Prison and Probation Service (HMPPS) in England and Wales. Each prison in HMPPS has a security classification, which ranges from A (maximum) to D (minimum). Each person in prison is allocated to the lowest security category appropriate to manage their risk, which is based on the risk of escape, harm to the public, ongoing criminality and violent behaviour that impacts other people in prison and disrupts the security of the prison. The categories are presented in Table 1 (HM Inspectorate of Prisons, 2020).

**TABLE 1:** Security classification of prisons.

Category	Classification
A	The highest category of security for prisoners whose escape would be highly dangerous to the public or a threat to national security
B	For prisoners who do not require the highest conditions of security, but for whom escape must be made very difficult
C	For prisoners who cannot be trusted in open conditions, but do not have the will or resources to make an escape attempt
D	For prisoners can be reasonably trusted to serve their sentence in open conditions, the lowest category of security

Purposive sampling was applied to recruit potential participants, to enable the identification of participants who could provide information‐rich discussions on their experiences related to the phenomena of interest (Palinkas et al., 2015). All potential participants were identified by the clinical practice leads within their university, who sent an email to their university email with information regarding the study. Nursing students were asked to contact the first author by email if they were interested in participating in the study. The inclusion criteria were university students studying one of three undergraduate nursing degrees (adult, mental health or learning disabilities), within two HEIs in England and had completed a primary care clinical placement within a prison.

### 3.3 Participants

All participants contacted participated in our study, which was an unexpected outcome, although reinforces the uniqueness of clinical placement in prison and the interest or even need of nursing students to discuss their experiences. Fourteen 2nd‐ and 3rd‐year nursing students participated, who were completing an undergraduate nursing degree to become a registered adult nurse (*n* = 4), learning disabilities nurse (*n* = 3) and mental health nurse (*n* = 7). The length of clinical placements ranged from 5 to 12 weeks. Most participants (*n* = 11) were required to submit a 500‐word expression of interest to obtain a clinical placement in prison. However, three participants were allocated a clinical placement in prison, due to COVID‐19 restrictions, as they did not have access to a car, and could not share a car with a community or primary care nurse (refer to Table 2).

**TABLE 2:** Overview of participants.

Participant	Discipline	Prison placement	HEI	Allocation of placement
1 (female)	Adult	CAT B2	South of England	Expression of interest
2 (female)	Adult	CAT A	South of England	Allocated by HEI
3 (female)	Adult	CAT A	South of England	Allocated by HEI
		CAT B2		
4 (female)	Adult	CAT A	South of England	Allocated by HEI
		CAT B2		
5 (female)	Learning Disabilities	CAT B1	Central England	Expression of interest
6 (female)	Learning Disabilities	CAT B1	Central England	Expression of interest
7 (female)	Learning Disabilities	CAT B1	Central England	Expression of interest
8 (female)	Mental Health	CAT B1	Central England	Expression of interest
9 (female)	Mental Health	CAT B1	Central England	Expression of interest
10 (female)	Mental Health	CAT B1	Central England	Expression of interest
11 (female)	Mental Health	CAT B1	Central England	Expression of interest
12 (male)	Mental Health	CAT C1	South of England	Expression of interest
13 (male)	Mental Health	CAT A	South of England	Expression of interest
14 (female)	Mental Health	CAT C2	South of England	Expression of interest

Abbreviation: HEI, Higher Education Institute.

Participants completed a clinical placement in one of five prisons, with a range of security classifications. The names of the five prisons within this study have been coded by their security classification and included one CAT A prison, two CAT B prisons (CAT B1 and CAT B2) and two CAT C prisons (CAT C1 and CAT C2). All prisons housed adult males with a capacity that ranged from 600 to 1200 people. Prisons housing women were not excluded; however, no prisons for women were within either university placement catchment area. Healthcare provision in each prison was provided by the NHS.

### 3.4 Data collection

Online semistructured interviews were completed via MS Teams between October and December 2021. The semistructured interview guide was developed from published literature (Sutherland et al., 2021; van de Mortel et al., 2017). The interview questions commenced with ‘why were you interested in applying for a clinical placement in a prison?’ and ‘can you describe the support and information you were provided from the university and/or the healthcare team in prison prior to commencing your placement?’. Nursing students' clinical experience was explored by ‘how was it different, if at all, providing care to someone who was in prison?’ and ‘can you explain the support your received during your clinical placement from the university and/or healthcare team?’. Finally, the impact of the clinical placement was explored by ‘can you describe the impact of your clinical placement on your you?’ and ‘how has this clinical placement influenced your perception of healthcare in prison?’. Follow‐up questions were applied to explore each participant's experience in‐depth. All interviews were conducted by the first author and lasted between 32 and 45 min.

### 3.5 Data analysis

All interviews were audio recorded and transcribed verbatim by the first author and checked by the second author. Data analysis was completed from an inductive phenomenological approach in adherence with the six phases of thematic analysis as described by Braun and Clarke (2022). The first phase, familiarization with the data, was commenced during the transcription process, and reading and re‐reading of the final transcripts, which was completed by J.B. and M.R. The following three phases coding, searching for themes and reviewing themes were completed simultaneously by J.B. and M.R. Codes were developed through notes and highlighting of direct quotes; this process was completed until no new codes were identified. When all codes were identified, these were explored and grouped to develop possible themes. The themes were reviewed by J.B. and M.R. through an in‐depth discussion, with reference to codes and returning to the raw data within each manuscript, following a reflexive and iterative process (Braun & Clarke, 2022). All authors were involved in the final defining and naming of the themes, and J.B. and S.S. wrote the analytic narrative. The presentation of direct quotes includes the language used by the nursing students who participated, such as the term ‘prisoner’, which is now considered to be a derogatory term for a person in prison (Tran et al., 2018). The nursing students applied this term as this was a term applied in the clinical placement they attended. However, the more frequently used term by nursing students was patient.

### 3.6 Rigour or trustworthiness

Rigour and trustworthiness of our study were addressed through the application of the four principles of credibility, transferability, dependability and confirmability identified by Lincoln and Guba (1985). Credibility was addressed and achieved throughout this study, to support an accurate depiction of nursing student's lived experience of a clinical placement in a prison setting. The context of the phenomena was understood by two authors, who have been involved in research within different prison settings and have firsthand experience of observations and the provision of care by primary care nurses within prison. Credibility and dependability were also addressed through peer debriefing with other members of the team (authors) to support in‐depth discussions. This process supported triangulation, as two authors completed the data analysis, and two authors cross‐checked the data to ensure the interpretations represented the lived experiences of participants. Transferability was addressed and achieved through purposive sampling, the research team had no previous contact with participants, either through teaching or being their academic assessor. Therefore, nursing students did not volunteer to participate to ‘please’ their lecturers or necessarily provide socially acceptable answers, supporting the provision of information‐rich data from their lived experiences. The data were continuously re‐read during data analysis, and the provision of direct quotes within the presentation of findings further supports transferability. Confirmability was achieved through the Heideggerian requirement of avoidance of a presuppositionless approach (Smith et al., 2009). Therefore, authors involved in data collection and analysis, engaged in the process of recognizing their preconceptions during these processes and as they arose, adhering to a Heideggerian approach of inductive phenomenology (Heidegger, 1962) to reduce potential bias. This was essential as the first two authors, as nurses, acknowledged their understanding of the provision of healthcare within prisons, which could influence their interpretation of nursing students' experiences.

### 3.7 Ethical approval

Ethical approval for this study was obtained from the Health, Education and Life Sciences Faculty Academic Ethics Committee at Birmingham City University on the 28th September 2021 (BROOKE/#9794/sub2/R(B)/2021/Sep/HELS FAEC). All participants received a participant information sheet and were provided with the opportunity to ask questions and have them answered. All participants were informed participation was voluntary, and if they did or did not participate, this had no impact on their ongoing studies or assignment grades. Following this process written informed consent was provided by each participant prior to their interview. All participants were aware support and counselling was available if required; the results of the study would be disseminated with direct anonymous quotes and they could withdraw their data for up to 2 weeks following their interview without consequences.

## 4 FINDINGS

The two overarching themes identified through a thematic analysis were ‘Shock’ and ‘Surprise’. Both themes capture how nursing students were trying to make sense of their experience of clinical placement in primary care in a prison setting. The first theme included shock due to the reality of prison; overwhelming emotional impact; and frequency and severity of self‐harm. The second theme included surprise due to (a) the need to work with prison officers; (b) recognizing and addressing their own preconceptions of people in prison and (c) the development of clinical knowledge, skills and becoming a nurse.

### 4.1 Theme 1: Shock

The overarching theme of shock was described by nursing students to make sense of their reactions to their clinical placement in a prison, subthemes included being shocked by the reality of prison, the overwhelming emotional impact, and the frequency and severity of self‐harm. Nursing students' experience of shock was linked to the level of support they described from their HEI placement team and their nurse mentor/manager within their clinical placement, which varied significantly. Several nursing students reported receiving information packs from the nurse mentor/manager prior to commencing their clinical placement: ‘from the prison we received two welcome packs, one that was sort of general prison rules, and what your first day would look like, and that was really helpful, the other explained healthcare provision and contact information of a nurse I was allocated’ (P10: MH: 17–20). Nursing students working with one mental health team in a category B security prison also had the opportunity to visit the prison prior to commencing their clinical placement, which was identified as very helpful: ‘The manager of the prison (healthcare) organised for me to visit the prior to starting … it just broke the ice, which I thought was the best thing, because on my first day I wasn't as nervous, as I felt I was prepared for it’ (P5: LD: 28–36). However, a few nursing students reported receiving no information from either their placement team or nurse mentor/manager.

#### 4.1.1 Subtheme 1: Shocked by the reality of prison

Nursing students were shocked by the reality of providing healthcare within a prison due to the level of security. The shock included how they were made to feel as though they had become a person in prison: ‘on entering the prison to go to healthcare you have to be checked, every door behind you has to be locked, moving forward just two steps, you have to unlock another door, and then lock it again, the stress was just too much, I felt like I was a prisoner’ (P4: AN: 33–44). Nursing students were also shocked by the vast range of objects that could not be brought into the prison, which further impacted their feeling of being a person in prison: ‘there were so many things that were unexpected and shocking, I couldn't write anything down whilst in there (prison) as I might not be allowed to take it out (the prison), and I couldn't bring anything back in I had previously written in there (prison), to be honest I felt like a prisoner’ (P13: MH: 31–36).

Nursing students were also shocked by the impact of security on their role as nurses: ‘The environment shocked me, for us to go out of our clinic, just to get out of our clinic, there were 10 doors, and for us to get to the prison building there were 20 doors, so you were under lock and key, you can't just walk around, so I think the environment was shocking and restricting’ (P1: AN: 47–51). However, the high level of security and feeling like a person in prison or a mental health patient under the section, supported nursing students in understanding the impact on people and patients on entering highly secure environments: ‘you really are in it with them (people in prison), you really are cut off from the world, so, to understand how anyone feels about being put into section or prison, where they cannot do anything, if you want to have an experience to understand how they feel, you need to work in the prison’ (P6: LD: 138–145).

#### 4.1.2 Subtheme 2: Shocked by the overwhelming emotional impact

Nursing students described the overwhelming emotional impact of entering prison for the first time and providing care to people in prison in a controlled environment: ‘I think overall, going for that first day was just overwhelming, I was just bombarded with information, and I couldn't take it all in…’ (P5: LD: 41–43). However, adult nursing students were more likely to find entering the prison as emotional: ‘My first day was emotional, I sat in my car at the end of my shift, and cried, I reflected on what we (nursing team) had been doing, which made me think about the patients and where they were, what the nurses have to go through to get to them, the conditions, the environment, all of that got to me’ (P1: AN: 42–46).

Nursing students were able to overcome their initial feelings of being overwhelmed during their clinical placement: ‘The nurses were incredibly lovely and supportive, but I did find it really overwhelming, and I am really glad it was a longer placement, because you need to be able to get comfortable within that environment, as everywhere was locked and they have to take you everywhere all of the time, you need to get used to’ (P7: LD: 42–47). Mental health nursing students discussed their anxiety on entering the prison, although their experiences of working in secure settings influenced their reactions to the security of a prison: ‘Going through security for the first time for me was quite reassuring as it was secure, although I have to move around with the nurse, I am working with…’ (P12: MH: 29–36).

#### 4.1.3 Subtheme 3: Shocked by the frequency and severity of self‐harm

Mental health and learning disabilities nursing students discussed they were not shocked by the act of self‐harm, as they were used to working with people who self‐harmed, but they were shocked by the severity and frequency: ‘I wasn't shocked by it, I work with people who self‐harm, but not as badly or as much as you saw within the prison, as there is a lot in there, I think that shocked me’ (P7: LD: 117–121). Mental health and learning disability students were also surprised why many of the people in prison self‐harmed: ‘I was surprised at how many people self‐harmed to gain what they wanted, they were all after single cells and stuff like that … I guess, it is that kind of environment, you are competing, not competing but surviving with 500 other men’ (P8: MH: 166–174).

However, adult nursing students were shocked by the act of self‐harm, as they identified they had not encountered self‐harm in their previous clinical placements. Adult nursing students also felt unprepared to understand self‐harm: ‘I thought self‐harm was people knocking their head against the wall, but there was a person we attended to, he had got hold of a can of coke, I don't know how, he opened the can and used it to harm himself’ (P1: AN: 171–179). Furthermore, adult nursing students were shocked by the role of a primary care nursing team as first responders to treating people in prison who had self‐harmed: ‘we (primary care nurses) were the first to go into self‐harm incidents and see the bleeding and stop the bleeding and send them to hospital if needed, no one spoke to us about self‐harm before we went into the prison’ (P1: AN: 171–179).

### 4.2 Theme 2: Surprised

The overarching theme of surprise was described by nursing students to make sense of their reactions to a clinical placement in prison. Subthemes included the surprise of working closely with prison officers, to provide healthcare to people in prison. Prison officers were described as supportive, although often lacked an understanding of health and focused on safety and the prison regime. Nursing students were surprised by their own preconceptions of people in prions, and the need to address these preconceptions. Finally, nursing students were surprised how the enclosed and secure environment supported them to become a nurses and look beyond these barriers.

#### 4.2.1 Subtheme 1: The need to work with prison officers

Nursing students acknowledged the support of prison officers, as they unlocked or called the individual for healthcare appointments. Prison officers also knew the individuals and if they posed any risk to healthcare professionals: ‘we report to the officers, so they can call the prisoner, if they are not on lockdown or in their cells, and the officers will always let us know if there were any security risks, the officers were very helpful’ (P12: MH: 58–66). Prison officers also supported nursing students to feel safe when interacting with individuals and enabled them to understand behaviours that were not tolerated: ‘being a young woman in prison was really daunting and if any of the prisoners said anything out of order to me, it would be a straight “no, you don't do that”’ (P7: LD: 74–80).

Nursing students also acknowledged some prison officers lacked an understanding of the impact of a mental health condition or a learning disability on the behaviour of people in prison: ‘I came across inmates with learning disabilities, and they (prison staff) didn't understand their behaviour was not challenging, it was due to their learning disabilities, which was heightened because they were in prison, the underlying cause was not for attention’ (P6: LD: 57–62). Although nursing students identified most prison officers were open to learning and implementing different initiatives to support people in prison: ‘you definitely come across some prison officers who are very stubborn and don't listen to the advice you are giving regarding someone's mental health’ (P14: MH: 60–66).

Nursing students identified working with prison officers to administer medications within the prison regime was challenging: ‘… as soon as the officers get the prisoner out they just want the prisoner to get the medication, so they can escort them back to their cells, and you are expected just to speed up, but when people are asking you to rush, can make people make mistakes’ (P13: MH: 121–127). However, nursing students acknowledged the important role of the prison officers in maintaining the prison regime and the safety of everyone in the prison, even though this sometimes impacted their ability to effectively administer medications: ‘our priority is patients' health and their priority is security and safety, and if they are understaffed, there isn't an officer to monitor medication rounds, then we have to stop the medication round, which impacts negatively on the patients, because they don't get their medication’ (P10: MH: 106–113).

#### 4.2.2 Subtheme 2: Recognizing and addressing preconceptions of prisons and people in prison

Nursing students frequently commenced their interviews with an exploration of their preconceptions of a clinical placement within a prison setting. Some nursing students discussed the need to keep an open mind, so their preconceptions did not impact on their experiences: ‘I didn't want to have any negative feelings about it (clinical placement in prison), so I went in with a positive and blank mind, ready to fill it in with what I learnt rather than fill it with any preconceptions, as then I may have been too scared to start’ (P3: AN: 19–21). Other nursing students admitted their preconceptions of people in prison impacted how they thought people in prison may behave and identified the need to behave differently within this clinical placement within a prison: ‘You need to be very self‐aware, you have to remember they (people in prison) have a lot of time on their hands, they will be watching you, taking notice, they are still criminals’ (P9: MH: 43–45).

Nursing students discussed the need to recognize their preconceptions, which influenced their decision to actively avoid knowing the offences committed by individuals, so they were able to provide non‐judgmental care: ‘I wasn't judging them, but I didn't know or want to know what brought them into prison, I know I am there to treat them as a nurse, we have to do our profession proud and we are not there to judge’ (P4: AN: 58–61). When nursing students were involved in providing mental health care and the nature of an individual's crime was discussed, nursing students struggled to remain non‐judgmental when these crimes involved children and the individual demonstrated no regret or guilt: ‘When a prisoner talked openly and in graphic detail about their crime I found that difficult when related to children, particularly when they showed no remorse, I found that really difficult, although I didn't treat them any differently, but I did find it very difficult’ (P7: LD: 154–158).

However, nursing students were also surprised by the respect received from people in prison, and began to reflect on the reasons why this occurred, ‘I think the general respect (from people in prison) was surprising, perhaps due to being female and not being part of the prison service, and working as a nurse, as a lot of the prisoners were always “hello miss” or “how are you miss”’ (P8: MH: 77–80). Nursing students also identified providing care for people in prison supported their understanding of some of the reasons why people may have become involved in crime, exploring both the influence of living in a deprived area and the reaction to an abuser: ‘I think working in prison opened my eyes, as I was surprised how much deprived backgrounds influence this (becoming a person in prison), as you don't get people from affluent backgrounds affected in the same way, you get people from poorer backgrounds and ethnically minority backgrounds, who have not had the opportunities that other people have had … and no one really asks them (person in prison) about the situation that occurred, for example, a lot of people have reacted to their abusers, and I don't know … if I was in that situation, would I be any different…’ (P9: MH: 54–61).

#### 4.2.3 Subtheme 3: Development of clinical knowledge, skills and becoming a nurse

A clinical placement within prison prompted nursing students to explore their current knowledge, skills, and abilities and how these had been further developed in the closed setting of a prison: ‘I have learnt a lot about risk management, reading someone's history and developing best care options, how to involve different teams, writing assessments, documentation I have learnt a lot, and I feel more confident in my abilities’ (P14: MH: 76–81). Nursing students also discussed how providing care within a prison setting supported the development of their skills: ‘I would see people (in prison) on a weekly basis, and that is where I felt I learnt the most, and developed my communication skills and relationships with patients, and my personal confidence, as it pushed me as it was a different way of nursing’ (P5: LD: 133–135).

A clinical placement in prison also supported nursing students in their personal development to become a nurse: ‘Being there (in prison), I began to think that I am a nurse, and it is my duty to care, so I can't be judgemental, I can't state I should not be here in prison, I need to develop my care’ (P1: AN: 76–81). Nursing students discussed the need to develop their skills to work with and treat all patients, even those who may demonstrate challenging behaviour: ‘Previously, I learnt a lot about being a nurse and the different skills I would need, but this (placement) was more about developing me, and how I should treat and care for people, and learnt how to deal with challenging behaviours, in prison challenging behaviours are very different’ (P4: LD: 138–144).

## 5 DISCUSSION

The lived experience of nursing students in a clinical placement in a healthcare setting in prisons in England, included the ‘Shock’ of the reality of the prison environment, the overwhelming emotional impact and the frequency and severity of self‐harm, and ‘Surprise’ due to working with prison officers, the need to recognize and address their preconceptions of prisons and people in prison, and how the clinical placement supported nursing students to become a nurse. Nursing students in our study identified clinical placements within a healthcare setting in a prison as secure, advantageous and distinctive. Previous studies have identified nursing students' positive feedback on clinical placement in prison and the development of community health understanding and competencies (Bouchaud et al., 2017; van de Mortel et al., 2017). Our study also identified a clinical placement in prison provided nursing students with a comprehensive and thought‐provoking nursing experience, which is demonstrated by their willingness, apart from one student, to return to healthcare provision in a prison environment as a registered nurse. Each theme will now be discussed in detail with relevant literature.

### 5.1 Theme: Shock

Nursing students' limited understanding of the unfamiliar and restrictive clinical environment within a prison, with strict protocols that impacted nursing practice, was a shock. The initial shock of the prison environment was emotionally overwhelming for some of the nursing students in our study, which caused anxiety. Pre‐placement anxiety before a clinical placement in a prison has been previously identified by nursing students, clinical psychology students and medical students (Abbott et al., 2020; Díaz et al., 2014; Needham & van de Mortel, 2020; Rohleder et al., 2006). Pre‐placement anxiety has been identified to increase when students feel unprepared for specific clinical placements (Bouchaud et al., 2017). Our study identified nursing students who had received documentation and attended a visit to the prison prior to commencing their clinical placement experienced less anxiety. However, there was significant variation in the documentation and support nursing students in our study received prior to commencing their clinical placements. The variation occurred across both HEIs and different healthcare teams within the different prisons.

The importance of an understanding of prison rules, safety regulations and how to interact with people in prison has been identified as necessary pre‐placement information (Brooke et al., 2022; Needham & van de Mortel, 2020). An example of supporting nursing students prior to clinical placement in prison is an insight day (Hunt et al., 2020), which was similar to the approach some of the nursing students in our study, who visited the prison prior to commencing their placement. Another different example is the use of simulation delivered within a HEI, which introduced nursing students to an interactive environment with scenarios that were realistic of providing healthcare within a prison (Díaz et al., 2014). Nursing students identified the simulation supported both a clinical orientation to the prison setting and an understanding of the prison population and their needs (Díaz et al., 2014).

An important aspect to be included in all education/induction programmes prior to a nursing student commencing a clinical placement within a prison is self‐harm. All nursing students in our study discussed the frequency and severity of self‐harm, which they were both shocked by and felt unprepared to support the people in prison who self‐harmed. This finding is not unexpected as the prevalence of self‐harm in the prison population is significantly higher than that of the general population (Hawton et al., 2014). Environmental factors of the prison may increase the risk of an individual engaging in non‐suicidal self‐harm, these include solitary confinement, disciplinary punishments, victimization, and poor social support (Favril et al., 2020). Barriers to preventing or managing self‐harm within prisons include a lack of staff with the confidence and training to support people in prison who are self‐harming (Hewson et al., 2022). All nursing students commencing a clinical placement in a prison require both knowledge and clinical skills to enable them to engage with and care for people in prison who have or may self‐harm.

### 5.2 Theme: Surprise

Nursing students within our study expressed surprise by the need to work closely with prison officers to provide healthcare to people in prison, but also to feel safe. Fear for personal safety was expressed by many of the nursing students in our study, which supports previous research findings (Hunt et al., 2020; van de Mortel et al., 2017). Prison officers supported nursing students to feel safe through their presence, knowledge of people in prison and the identification of and stopping of inappropriate behaviour and/or the language of people in prison. Similar to previous studies, prison officers were essential for nursing students to feel safe within prison and when interacting with people in prison (Bouchaud et al., 2017; Sutherland et al., 2021). Nursing students have even identified they felt safer in prison than in an emergency department on a Saturday night due to the presence of prison officers (Sutherland et al., 2021).

The provision of mental health services for people in prison was identified by the nursing students in our study as problematic due to prison officers' lack of understanding of mental health conditions. The training programme to become a prison officer varies across countries, including the length, depth, and place of training (Ryan et al., 2022). In England and Wales, the prison officer training programme occurs over 10 weeks and includes how to look after people in custody, search and security procedures, and de‐escalation techniques (HM Prison and Probation Service, 2024). The focus of the training programme highlights the need for continued professional development of prison officers, especially with a focus on mental health, dementia, and substance misuse (Brooke & Rybacka, 2020). A focus on mental health training within the prison officer training programme is essential as this has been associated with a positive change in prison officer's perceptions of mental health conditions (Kois et al., 2020). Nursing students in our study identified most prison officers were willing to learn and implement recommendations to support people in prison with their mental health.

Nursing students in our study identified the difficulty of working within the prison regime, which was prioritized by prison officers over the provision of healthcare. A particular concern of the nursing students in our study was the safe administration of medications. Previous research has identified the negative impact of the prison regime on the safe administration of medications (Magola‐Makina et al., 2022). The negative impact was caused by the restricted time to complete medication administration and the reduced or lack of capacity of prison officers to manage and supervise people in prison when queuing for their medications (Magola‐Makina et al., 2022). Nursing students in our study also identified the need to rely on prison officers to escort people in prison to healthcare appointments, so appointments were sometimes missed. The presence or close proximity of a prison officer during an appointment has also been recognized to have a negative impact due to creating an imbalance of power, invading the privacy and confidentiality of the person in prison, and limiting the time of the appointment, by rushing or ending the appointment early (Edge et al., 2020).

Nursing students in our study were surprised by the need to address their preconceptions of prisons and people in prison. The need for nursing students to identify their own negative stereotypes of people in prison prior to clinical placement has been previously recognized (Hunt et al., 2020; Terblanche & Remier‐Kirkham, 2020). However, preconceptions and negative stereotypes of people in prison were often based on media portrayals of this population (Bouchaud et al., 2017; Hunt et al., 2020). As within our study, previous studies have identified understanding the real‐life stories of people in prison, supported nursing students in our study to feel empathy for some people in prison and supported them to relate to these individuals (Hunt et al., 2020; Terblanche & Remier‐Kirkham, 2020). The real‐life stories also supported nursing students in our study to understand the impact of deprived communities on the lives of individuals, and to be able to visualize the impact of social determinants on health.

Finally, nursing students in our study were surprised how the enclosed, secure, and restrictive environment of a prison supported them to become a nurses and provide care despite these barriers. A clinical placement in a prison has been identified to support the development of nursing students' self‐assurance, ability to provide unbiased care for patients who may exhibit challenging behaviours, as well as high‐quality care to a vulnerable population with low health literacy (Bouchaud et al., 2017; Needham et al., 2023; Sutherland et al., 2021). The unique environment of providing healthcare within a prison supported the nursing students in our study to address their biased preconceptions and develop their clinical understanding and skills to provide unjudgemental care and support to a vulnerable population.

## 6 LIMITATIONS

One limitation of our study was the recruitment of nursing students from only two HEIs within England. One HEI offered a clinical placement in prison following a process of expressing an interest, while one HEI allocated nursing students, which may have impacted the results of our study. Other variables may have impacted our results such as the demographic variables of students and their year of study. A further important consideration is neither of the HEIs provided education on the health and social care needs of people in prison or provided healthcare in a prison, which may not be representative of other HEIs in England or internationally. However, the strength of our study is the inclusion of five different prisons where nursing students completed their clinical placements, although these were all male prisons.

## 7 CONCLUSION AND RECOMMENDATIONS

Our research reinforces the need for a programme to support the preparation and orientation of nursing students before commencing a clinical placement in prison. The programme needs to be collaboratively provided by HEIs and healthcare providers and includes the health and social care needs of people in prison, the provision of healthcare within a prison, and the rules and regulations of a prison. An important element of the programme is the exploration of nursing students' preconceptions of people in prison and prisons, to ensure these are addressed. Clinical placement in healthcare in a prison enables nursing students to recognize and address their negative perceptions towards diverse and disadvantaged populations and provides a unique opportunity to develop and enhance their clinical knowledge and skills, and to develop as a nurse.

## AUTHOR CONTRIBUTIONS

All authors have agreed on the final version and meet at least one of the following criteria (recommended by the ICMJE): substantial contributions to conception and design, acquisition of data, or analysis and interpretation of data; drafting the article or revising it critically for important intellectual content.

## FUNDING INFORMATION

This research received no specific grant from any funding agency in the public, commercial, or not‐for‐profit sectors.

## CONFLICT OF INTEREST STATEMENT

All authors declare they have no conflict of interest related to this study.

### PEER REVIEW

The peer review history for this article is available at https://www.webofscience.com/api/gateway/wos/peer‐review/10.1111/jan.16355.

## ETHICS APPROVAL STATEMENT

Ethical approval was obtained from the Birmingham City University, Health, Education and Life Sciences Faculty Ethics Committee (BROOKE/#9794/sub2/R(B)/2021/Sep/HELS FAEC).

## PARTICIPANT CONSENT STATEMENT

All participants provided informed consent.

## Supporting information


Data S1.

## Data Availability

The proportion of the data that support the findings of this study are available on request from the corresponding author. The data are not publicly available due to privacy or ethical restrictions.
